# Does a whole plant conservation gradient exist within a subtropical broadleaved evergreen forest?

**DOI:** 10.3389/fpls.2024.1481323

**Published:** 2024-12-11

**Authors:** Ailian Fan, Xue Wang, Xiaojun Yan, Tingting Chen, Qi Jiang, Linqiao Jia, Weiwei Wang, Decheng Xiong, Jinxue Huang, Guangshui Chen

**Affiliations:** ^1^ Key Laboratory of Humid Subtropical Eco-geographical Process of Ministry of Education, School of Geographical Sciences, Fujian Normal University, Fuzhou, China; ^2^ Fujian Sanming Forest Ecosystem National Observation and Research Station, Fujian Normal University, Fuzhou, China

**Keywords:** acquisition strategy, conservation strategy, functional trade-off, interspecific variation, leaf-root coordination, plant economics spectrum, subtropical broadleaved evergreen forest

## Abstract

The coordination between leaf and root traits is crucial for plants to synchronize their strategies for acquiring and utilizing above- and belowground resources. Nevertheless, the generality of a whole plant conservation gradient is still controversial. Such testing has been conducted mainly among communities at large spatial scales, and thus evidence is lacking within communities. This is noteworthy because factors that influence leaf and root trait variation differ across scales. Here, we measured pairs of analogous leaf and first-order root traits, including morphological (leaf thickness (LT) and root diameter (RD), leaf mass per unit area (LMA) and specific root length (SRL), and leaf and root tissue density (LTD and RTD)) and chemical traits (carbon (C) and nitrogen (N) concentrations in leaf and root tissues), on the same plants from 60 woody species within a subtropical broad-leaved evergreen forest. The trade-off patterns in and correlations between leaf and root traits were examined using (phylogenetic) principal component analysis and correlation analysis. Our results revealed two dominant dimensions of leaf trait variation, the leaf economics spectrum (LES) and the LT-LTD trade-off axis. Variations in root traits were mainly accounted for by a two-dimensional root economics space (RES) (i.e., root conservation gradient (RTD-RN) and root collaboration gradient (RD-SRL)). The LES and root conservation gradient were correlated and could be integrated into one whole plant conservation gradient, independent of the root collaboration gradient and the leaf LT-LTD trade-off dimension. Leaf and root N concentrations correlated positively, independent of phylogeny, whereas analogous leaf and root morphological traits varied independently of each other. These results support the existence of a whole plant conservation gradient, but also highlight a complex integration of multiple above- and belowground adaptive strategies of plants within a forest community, which offer new insight into ecological trade-offs, species coexistence and community assembly in the forest ecosystem.

## 1 Introduction

The variations and covariations of functional traits in plants can reflect their ecological strategies, thereby, facilitate an understanding of community attributes and ecosystem functions in response to environmental change (Díaz and Cabido, 2001; Díaz et al., 2016; Maynard et al., 2022). So far, a “fast-slow” whole plant economics spectrum (PES) has been proposed to reflect the coordinated strategies of plants in dealing with multiple forms of stress in addition to resource limitation (Freschet et al., 2010; Reich, 2014). Central to the PES hypothesis is the idea that rates of water and nutrient uptake should function in concert with those of carbon (C) acquisition for optimal plant growth (Reich, 2014). Because leaves and roots are the plant organs primarily responsible for acquiring above- and belowground resources, respectively, they have received significant attention in trait coordination research (Wright et al., 2004; Roumet et al., 2016). So far, however, evidence for coordination between analogous leaf and root traits is inconclusive. A large number of studies have shown that nitrogen (N) (Li et al., 2010; Wang et al., 2017; Hu et al., 2019; Yang et al., 2021; Weigelt et al., 2021) and phosphorus (P) concentrations (Holdaway et al., 2011; Geng et al., 2014; Yang et al., 2021; Weigelt et al., 2021) correlate positively between leaves and roots, thus supporting the nutrient portion of the PES hypothesis. With morphological traits, however, the results are mixed. For example, the specific leaf area–specific root length (SLA-SRL) relationship has been reported as positive (Zhou et al., 2018; Erktan et al., 2018), negative (Messier et al., 2017), and nonsignificant (Craine et al., 2001; Fort et al., 2013; Hajek et al., 2013; Simpson et al., 2016; Wang et al., 2018a; Shen et al., 2019). Tissue density and organ thickness were also found to have poor or no correlation between leaves and roots (Kembel and Cahill, 2011; Shen et al., 2019). These diverse relationships between analogous leaf and root traits imply that a single “fast-slow” PES fails to explain all above- and below-ground plant strategies.

Leaves and roots might encounter contrasting selection pressures and constraints throughout their evolutionary processes (Fortunel et al., 2012; Freschet et al., 2013). Leaves are primarily optimized to maximize light capture and CO_2_ fixation (Poorter et al., 2009), thus leading to a relatively consistent trade-off pattern in leaf traits. A one-dimensional leaf economics spectrum (LES) has been observed at global (Wright et al., 2004), regional (Wang et al., 2017), and local scales (Yang et al., 2021). However, because roots encounter more complex environmental conditions, need to uptake different resources and are generally symbiotic with mycorrhizal fungi (Weemstra et al., 2016; Ma et al., 2018), they might exhibit a higher degree of variation and more diverse trait tradeoffs. Recently, a two-dimensional root economics space (RES) has been proposed (Bergmann et al., 2020), that is, the root collaboration gradient (root diameter (RD)-SRL axis) and the conservation gradient (root tissue density (RTD)– root nitrogen concentration (RN) axis). It has been supported by global- (Bergmann et al., 2020; Carmona et al., 2021), regional- (Wang et al., 2018b), and local-scale studies (Han et al., 2022). After integrating the above- and belowground traits of 2510 species from around the world, Weigelt et al. (2021) proposed that plant traits are multidimensional in general, not just root traits, and that only the above- and belowground conservation gradients may be correlated, representing a whole plant conservation gradient. Nevertheless, the basic controversy on the generality of such a whole plant conservation gradient is still unresolved. For example, Carmona et al. (2021) reached the opposite conclusions, on the basis of data largely overlapping with Weigelt et al. (2021), that a common axis of LES and fine-root economic traits was not found. Part of the controversy might arouse from the quality of data used. In some studies, not all the data used are measured, but, for example, using a gap-filled dataset (Carmona et al., 2021); some studies used data of leaf and root traits not measured on the same plants (e.g., using the TRY database which in general are based on different plants). As plant traits vary largely across different scales and from individuals to individuals within a species, using data not strictly measured on the same plants might bring some uncertainty in examining the generality of a whole plant conservation gradient.

**Figure 1:**
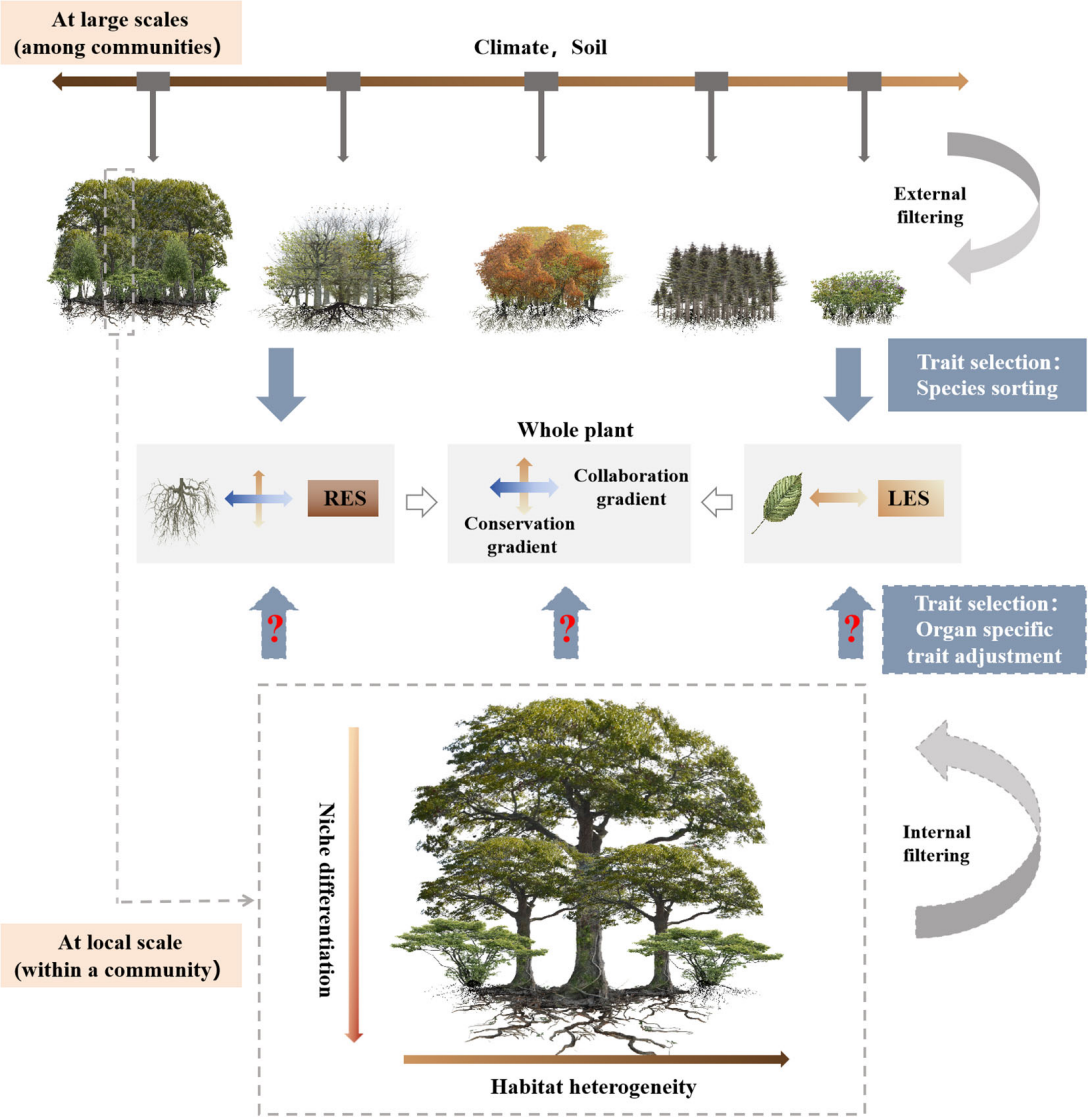
A conceptual framework explaining the variation of plant functional traits at different scales. LES, leaf economics spectrum; RES, root economics space.

The factors driving covariation among plant traits generally differ across spatial scales (Messier et al., 2017). At large scales, plant traits are selected by species sorting at the level of individual plants, and this process is primarily influenced by external filtering factors such as climate and soil conditions (Violle et al., 2012; Joswig et al., 2022) (**Figure 1**). Currently, an increasing number of studies have focused on the covariation of leaf and root traits across global or regional environmental gradients, including soil nutrient availability (Kramer-Walter et al., 2016; de la Riva et al., 2018; Wang et al., 2018a), drought severity (Liu et al., 2010; Butterfield et al., 2017), and the elevation gradient (Weemstra et al., 2022), etc. However, it remains unclear whether findings from these large-scale studies are also true at a local scale. Within a forest community, internal filtering mechanisms such as resource competition or habit heterogeneity primarily influence species coexistence and drive adjustments in plant traits at the organ level (Violle et al., 2012; Silvertown, 2004) (**Figure 1**). Light availability may serve as an important factor in distinguishing aboveground ecological niches within forest communities (Shen et al., 2019), thereby making it an important driver of leaf trait variation within a forest community. However, roots might encounter large interspecific competition within the same forest community and significant microscale heterogeneity in soil resources and the environment. This suggests that niche differentiation may be greater for roots than for leaves (Read et al., 2017). Therefore, examining the correlations between leaf and root traits within a forest community may help to confirm whether trait covariation patterns are spatial scale dependent both above- and belowground, as well as to reveal the life history strategies and trade-offs that govern plant species coexistence within a community (Holdaway et al., 2011; Kandlikar et al., 2022). Past within-community trait studies have focused predominantly either on leaves (Bin et al., 2022) or on roots (Han et al., 2022). Even when leaf and root traits were studied together, these investigations were generally limited to a few species in the community, such as the understory species (Burton et al., 2017; Shen et al., 2019). Thus, the relationship between leaf and root traits within a community remains unclear (Yang et al., 2021).

Roots have a complex branching system, and the structure and function of roots vary significantly at different orders (Pregitzer et al., 2002). The classification of fine roots remains an important prerequisite in the study of root structure and function within the context of RES. First order roots are the most distal roots with the most rapid turnover rate and the highest metabolic activity. They are considered most functionally comparable to leaves, which are another resource acquisition organ (Guo et al., 2008; Wang et al., 2017). However, the majority of previous studies regarding root-leaf trait correlation have focused on diameter-defined fine roots (<2 mm in diameter) (de la Riva et al., 2018; Delpiano et al., 2020; Gao et al., 2023). Only in rare cases have they investigated the functionally defined absorptive or first-order roots (Bergmann et al., 2017; Shen et al., 2019).

Subtropical evergreen broad-leaved forests are characterized by high community complexity, with very high species richness and a diverse array of functional traits (Bin et al., 2022; Zhang et al., 2011). The primary objective of this study was to examine the dimensionality in and covariation between leaf and first-order root functional traits among different species in a subtropical evergreen broad-leaved forest, based on data of paired leaf and root traits measured on the same individual plants. Based on the concept illustrated in **Figure 1**, we hypothesized that: (1) a one-dimensional LES and a two-dimensional RES should exist within a community of the subtropical evergreen broad-leaved forest; and (2) the LES should correlate with the root conservation gradient, representing a whole plant conservation gradient.

## 2 Materials and methods

### 2.1 Study site

This study was conducted in Wanmulin National Reserve, Jian’ou, Fujian, China (27°02′28″-27°03′32″N, 118°02′22″-118°09′23″E). This area has a subtropical monsoon climate, with an average annual temperature of 18.8°C and a frost-free period spanning over 277 days. The mean annual precipitation amounts to 1673 mm and primarily occurs during the period from April to June. The mean annual relative humidity is 80%. The terrain consists of low mountains and hills having an altitude of 234-556 m. The subtropical evergreen broad-leaved forest studied here covers an area of 1.89 km^2^, but it exhibits remarkable species richness, with a total of 1205 vascular plant species representing 581 genera and 161 families (30 families of ferns, 7 families of gymnosperms, and an impressive array of angiosperm taxa comprising 124 families) (Zhu et al., 1997). The most representative plant families in this forest are Fagaceae, Magnoliaceae, Lauraceae, Symplocaceae, and Theaceae. Dominant species include *Cinnamomum camphora*, *Castanopsis carlesii*, *Phoebe zhennan*, *Castanopsis faberi*, etc.

### 2.2 Leaf and root sampling and trait measurement

During June and July 2018, based on the results of the plant community biodiversity survey in this Nature Reserve, 60 dominant woody tree species (**Supplementary Table S1**) were selected based on the dominance in tree and shrub layers in a representative 40 m × 80 m plot within the forest. For each species, three individuals with similar size (diameter at breast height or ground diameter) were selected, and then the paired leaf and root samples were collected from each individual. Five analogous leaf and root traits were measured, 10 functional traits in total (**Table 1**), including 6 core traits (LMA, LN, RD, SRL, RTD, RN) which were commonly used to define the LES and RES axes (Weigelt et al., 2021). Fifty fully expanded (current year) and undamaged leaves were collected from the upper crown of each individual. Leaf thickness (LT) (mm) was measured with a digital display vernier caliper (with an accuracy of 0.02 mm), and leaf area was quantified using a leaf area meter (LI-3000C, USA). Leaves were then dried at 65 °C for at least 72 h to a constant weight and weighed. Leaf area and dry mass were then used to calculate leaf mass per unit area (LMA) (g m^−2^). Leaf tissue density (LTD) (g cm^-3^) was calculated as leaf dry mass/(leaf area × leaf thickness). Total leaf C and N concentrations (mg g^-1^) were determined using an elemental analyzer (Vario EL III, Elementar, Germany).

**Table 1:** List of the 10 functional traits and their ecological functions.

Organ	Trait	Abbreviation	Unit	Functions	Core trait? (Y/N)
Leaf	Leaf thickness	LT	mm	Resource capture and defense	No
	Leaf mass per unit area	LMA	g·m^-2^	Resource capture and defense	Yes
	Leaf tissue density	LTD	g·cm^-3^	Resource capture and defense	No
	Leaf carbon content	LC	mg·g^-1^	Resource capture and defense	No
	Leaf nitrogen content	LN	mg·g^-1^	Resource capture	Yes
Root	Root diameter	RD	mm	Transport, structure, and defense	Yes
	Specific root length	SRL	m·g^-1^	Resource capture	Yes
	Root tissue density	RTD	g·cm^-3^	Transport, structure, and defense	Yes
	Root carbon content	RC	mg·g^-1^	Resource capture and defense	No
	Root nitrogen content	RN	mg·g^-1^	Resource capture	Yes

Root samples were collected according to the method described by Guo et al. (2008). The main root branch near the tree stem was partially excavated just enough to trace its lateral root clusters. Then root clusters with intact branch orders were cut from the main lateral woody roots by extracting an intact soil block (about 20×20×20 cm) and immediately transporting it to the laboratory for further morphological and chemical analyses. In the laboratory, after careful cleaning of adherent soil particles and organic matter, the root branching clusters were dissected into different branch orders following the description of Pregitzer et al. (2002), being kept moist with deionized water throughout. Here, we focused only on first-order roots. Root segments of each order were scanned by the Epson scanner, and the root diameter, length, and volume were measured using Win RHIZO (Pro 2009b). All scanned roots were then oven-dried at 65°C for 72 h and weighed to determine their root dry matter. SRL (m g^-1^) was calculated as root length/root dry weight, and RTD (g cm^-3^) was calculated as root dry weight/root volume. Total root C and N concentrations (mg g^-1^) were determined using an elemental analyzer (Vario EL III, Elementar, Germany).

### 2.3 Construction of the phylogenetic tree

Species name and taxonomic nomenclature were standardized and corrected according to the Plant List (http://www.theplantlist.org) using the “plantlist” package (Zhang, 2018). The phylogenetic tree was constructed based on the comprehensive angiosperm species-level phylogeny of Zanne et al. (2014), updated by Qian and Jin (2016), using the “V.PhyloMaker” package (Jin and Qian, 2019). The mega-tree implemented in “V.PhyloMaker” (i.e. GBOTB. extended.tre), which included 74533 species and all families of extant vascular plants, was the largest dated phylogeny for vascular plants to date (Jin and Qian, 2019). All 60 species were included in this phylogenetic tree (**Supplementary Figure S1**).

### 2.4 Data analyses

All data analyses in this study were conducted at the species level. Thus, species mean trait values are used throughout. All trait data was log10-transformed to meet the assumption of normality.

The mean value (Mean), extreme values (Min and Max), median value (Median), and coefficient of variation (CV) were calculated for each trait using the “stat.desc” function in the “pastecs” package. To assess the phylogenetic conservatism in both above-and belowground traits, we calculated phylogenetic signal in all traits by performing Pagel’s *λ* testing (Pagel, 1999). Pagel’s *λ* provides a reliable metric for discriminating between complex models of trait evolution (Münkemüller et al., 2012). Pagel’s *λ* ranges from 0 to 1. A larger value indicates greater phylogenetic conservatism for the given trait. Significance was tested by comparing standardized contrast variables to random values obtained by shuffling trait data across the tips of the tree 999 times. The significance test was performed using the “phylosig” procedure in the “phytools” R package.

We used the “principal” function in the “psych” package (Venables and Ripley, 2002) to perform principal component analysis (PCA) on leaf, first-order root, and whole plant traits (leaf and root traits combined). Phylogenetic principal component analysis (pPCA) was also performed using the “phyl.pca” function in the “phytools” package to identify the dominant dimensions of trait covariation (Revell, 2012). pPCA has been widely used in the determination of independent axes of functional specialization (e.g., Liu et al., 2015; Wang et al., 2018b; Montesinos-Navarro et al., 2020). Student’s t-tests were used to assess differences between growth forms (shrubs and trees). These tests were performed using the “ggpubr” package.

To evaluate the impacts of phylogenetic autocorrelation on trait relationships between leaves and roots, we compared generalized least squares (GLS) regressions with phylogenetic generalized least squares (PGLS) analyses. The latter method accounted for evolutionary association among species and yielded unbiased regression coefficients and significance levels (Revell, 2010). For phylogenetic analyses, we first assessed different phylogenetic correlation structures (Brownian, Martin’s, and Pagel’s), and then we selected the best method by comparing Akaike’s information criterion (AIC). These preliminary analyses showed that Pagel’s *λ* was the best phylogenetic correlation structure (lowest AIC, **Supplementary Table S6**). We thus applied this methodology, in which phylogenetic regression was performed with a phylogenetic tree whose internal branches were multiplied by *λ*, leaving the tip branches at their original length, in all tests of the trait relationships in our study (Revell, 2010). Here, *λ* was estimated with maximum likelihood using the “gls” function from the R package “nlme”.

All statistical analyses were performed in the R4.1.2 statistical platform (R Core Development Team, http://www.r-project.org/), and all results were visualized using the “ggplot2” package.

## 3 Results

### 3.1 Multivariate variation of leaf and first-order root traits

Among leaf traits in the subtropical evergreen broad-leaved forest, CV was highest for LT (61.8%) and lowest for LC (7.8%). Among root traits, SRL displayed the highest CV (75.1%) and RC showed the lowest (5.1%) (**Table 2**). The CVs were generally higher for morphological traits than for chemical traits (**Table 2**). Among leaf traits, LC (*λ* = 0.89, *p* < 0.001) exhibited significant, and LN (*λ* = 0.766, *p* = 0.058) displayed marginally significant phylogenetic signal, but no phylogenetic signal was found for any leaf morphological trait. In contrast, all root traits, except for RC, showed significant phylogenetic signals (**Table 2**).

**Table 2:** Leaf and first-order root traits and their phylogenetic signal in 60 tree species in a subtropical evergreen broad-leaved forest.

Organ	Trait	Minimum	Maximum	Median	Mean ± SE	Coefficient of Variation (%)	Pagel’s *λ*
Leaf	LT (mm)	0.12	1.16	0.32	0.40± 0.03	61.8	0
	LMA (g·m^-2^)	52.00	217.00	110.00	116.22 ± 4.25	28.3	0.290
	LTD (g·cm^-3^)	0.09	0.98	0.32	0.38 ± 0.03	56.6	0
	LC (mg·g^-1^)	356.85	509.5	456.38	451.67± 4.55	7.8	0.890^***^
	LN (mg·g^-1^)	10.76	33.49	16.93	17.44± 0.55	24.5	0.766 (*p*=0.058)
First-order root	RD (mm)	0.19	0.63	0.36	0.37± 0.01	25.2	0.244^*^
	SRL (m·g^-1^)	13.12	190.13	34.62	43.69 ± 4.23	75.1	0.666^*^
	RTD (g·cm^-3^)	0.07	0.83	0.29	0.33 ± 0.02	52.5	0.763^*^
	RC (mg·g^-1^)	346.3	486.1	434.97	433.76± 2.85	5.1	0.115
	RN (mg·g^-1^)	5.54	30.19	12.55	13.78 ± 0.72	40.3	0.473^***^

^*^, *p* < 0.05; ^***^, *p* < 0.001. Trait abbreviations are the same as in **Table 1**.

**Figure 2:**
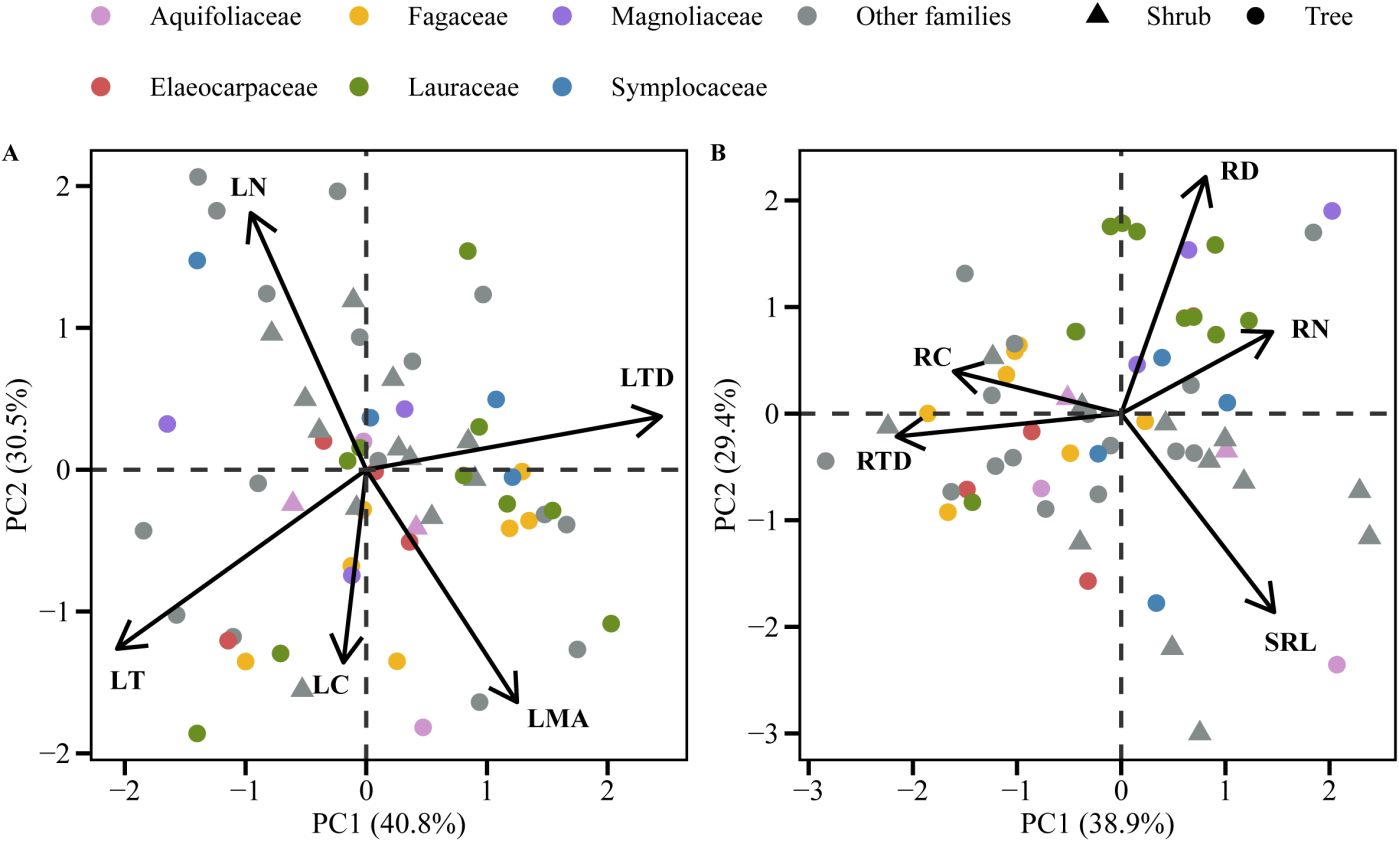
Phylogenetic principal component analysis (pPCA) (leaves **(A)** and first-order roots **(B)**) of functional traits in 60 woody species in a subtropical evergreen broad-leaved forest. Trait abbreviations are in **Table 1**.

The first two principal components of both pPCA and PCA explained more than 70% of the total variation in leaf traits (**Figure 2A**; **Supplementary Figure S2A**; **Supplementary Table S2**). The PC1 axis was strongly and positively correlated with LTD and negatively correlated with LT, thus reflecting an adaptive trade-off in leaf construction in response to light heterogeneity within the forest community (**Figure 2A**; **Supplementary Figure S2A**). The PC2 axis correlated positively with both LN and negatively with LMA in pPCA, and the relationships had the opposite signs in PCA. These reflect the dimension of the leaf economics spectrum (**Figure 2A**; **Supplementary Figure S2A**). The results of root functional trait pPCA and PCA both showed two dimensions of RES (**Figure 2B**; **Supplementary Figure S2B**; **Supplementary Table S3**). The first axis was positively correlated with RN and negatively correlated with RTD, thus representing the root conservation gradient. The second PCA axis correlated positively with RD and negatively with SRL, thereby representing the root collaboration gradient (**Figure 2B**; **Supplementary Figure S2B**). Shrubs were mainly distributed at the “do-it-yourself” end of the root collaboration gradient (mainly located on the high SRL side) (**Figure 2B**; **Supplementary Figure S2B**). Trees and shrubs differed significantly in their PC2 axis scores (**Supplementary Figure S3D**, *p* < 0.01), with shrubs having a significantly higher SRL than trees (**Supplementary Figure S4G**, *p* < 0.05).

### 3.2 Covariation between leaf and first-order root traits

**Figure 3:**
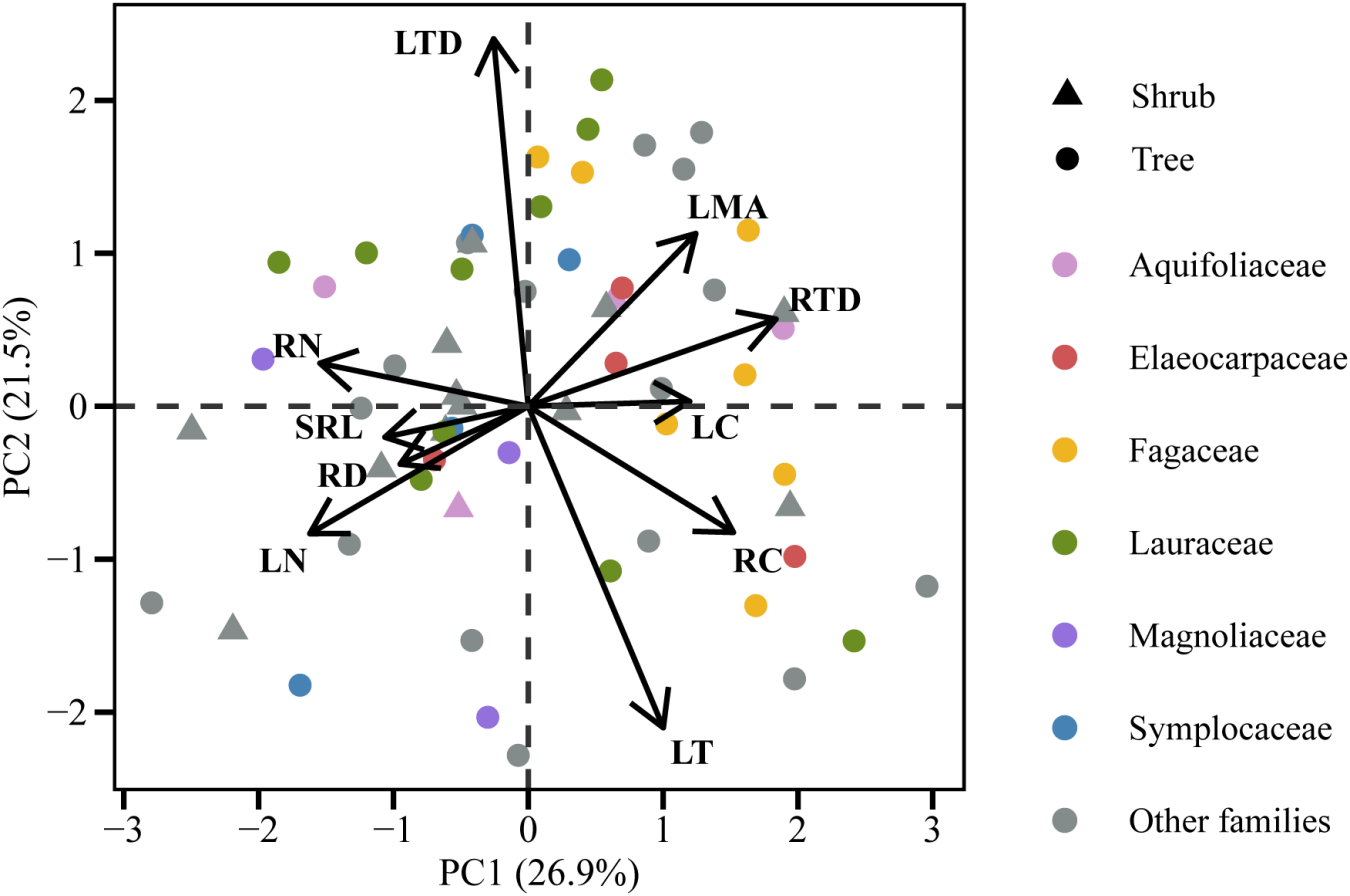
Phylogenetic principal component analysis (pPCA) for 10 leaf and first-order root traits of 60 tree species in the subtropical evergreen broad-leaved forest.

The pPCA and PCA analyses of leaf and root traits combined (10 traits in total) revealed that the first principal component represented the whole plant conservation gradient, with species exhibiting higher RN and LN at one end of the axis, and species displaying higher LMA, RC, and RTD positioned at the opposite end (**Figure 3**; **Supplementary Figure S5**; **Supplementary Tables S4** and **S5**). The LT-LTD trade-off axis and the root collaboration gradient (SRL-RD) were independent of the whole plant conservation gradient and were located in the second and third principal components, respectively (**Figure 3**; **Supplementary Figure S5**; **Supplementary Tables S4** and **S5**). The PCA and pPCA analyses of the six core leaf and root traits also showed that RN, LN, LMA, and RTD were predominantly aligned with the whole plant conservation gradient on the first axis, while the second axis represented the root collaboration gradient (**Supplementary Figure S6**).

**Figure 4:**
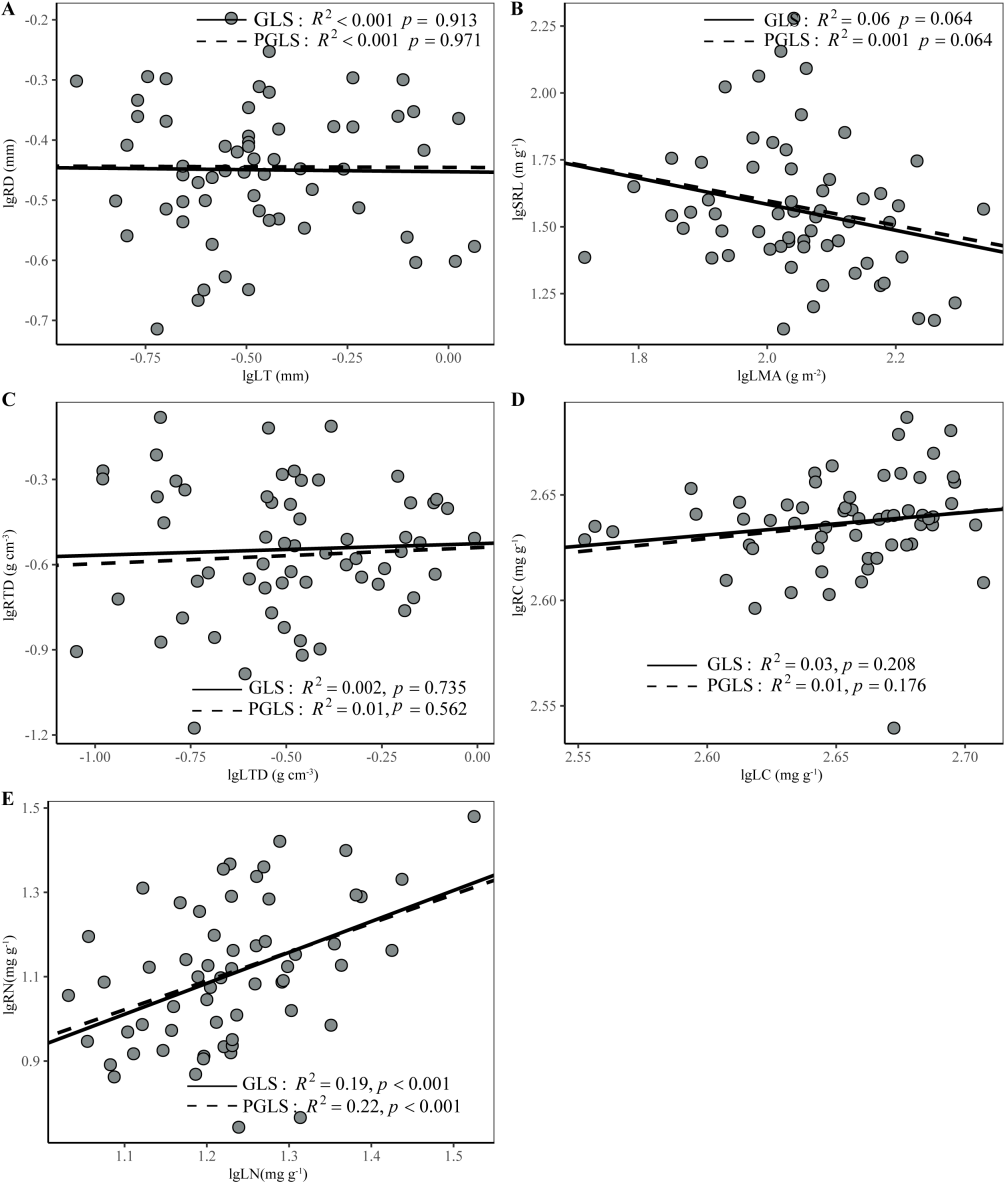
Relationships between analogous leaf and first-order root traits in 60 woody species in the subtropical evergreen broad-leaved forest calculated using generalized least squares (GLS) and phylogenetic generalized least squares (PGLS) methods **(A–E)**. Model results of the two methods are given in **Supplementary Table S7**. All trait data was log10-transformed before analysis. All trait data was log10-transformed before analysis. Trait abbreviations are the same as in **Table 1**.

No correlation was found between any other pair of analogous leaf and root traits, that is, between LMA and SRL, LT and RD, LTD and RTD, or LC and RC (**Figures 4A–D**, p > 0.05). On the same whole plant conservation gradient, LMA and RTD had a weakly positive correlation (**Supplementary Figure S7A**). However, there was a positive relationship between leaf and root N concentrations, which persisted even after adjusting for phylogenetic relatedness (**Figure 4E**, *p* < 0.001).

## 4 Discussion

### 4.1 Evidence of LES and RES within the subtropical broadleaved evergreen forest

**Figure 5:**
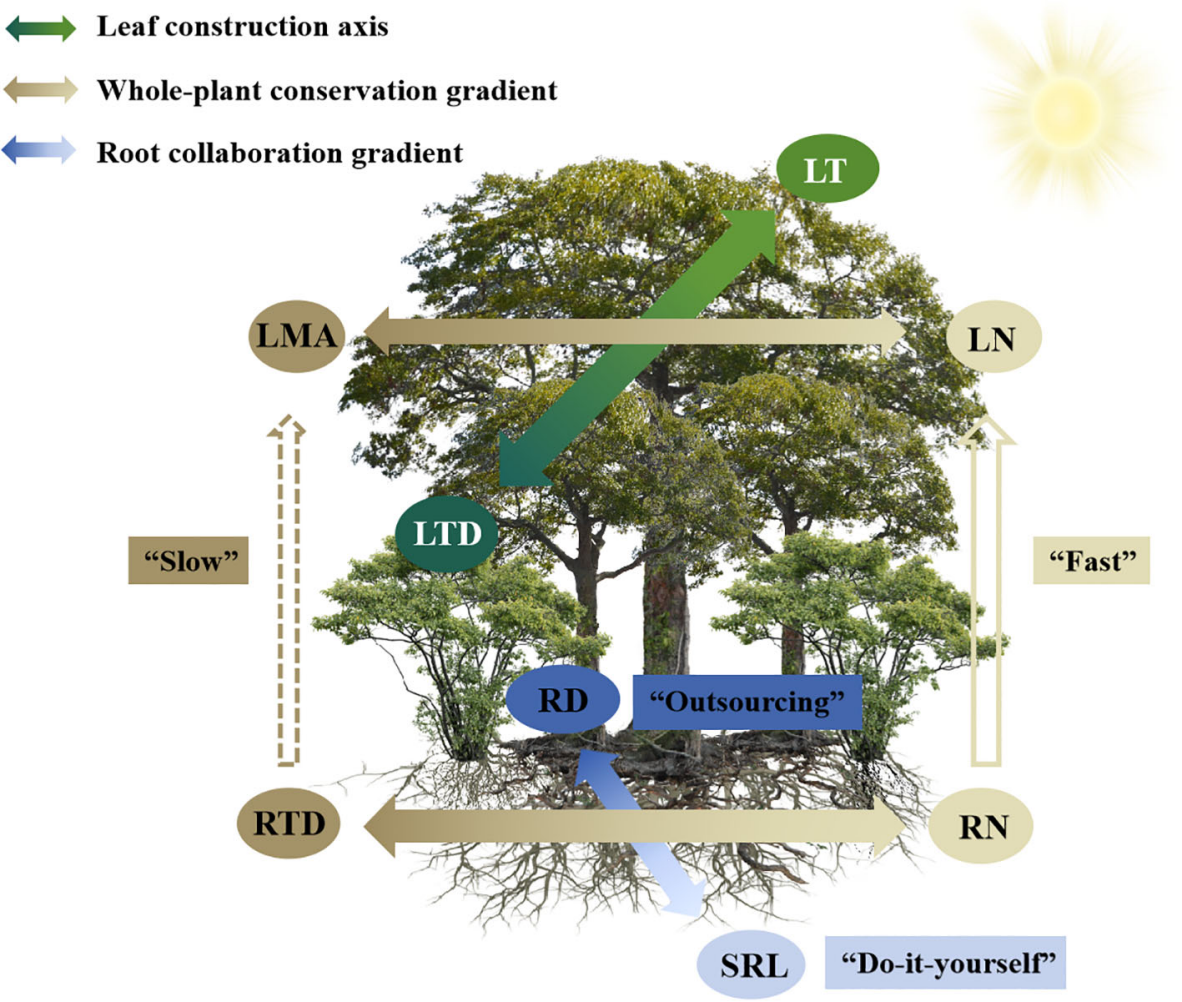
A conceptual framework explains the construction of plant trait dimensions (including leaves and first-order roots) within a subtropical evergreen broadleaved forest. The common leaf economics spectrum (LES) and two-dimension root economics space (RES) are evident within this community. An LT-LTD trade-off dimension is also obvious, representing an adaptation of leaf construction to light heterogeneity. Leaf and root conservation gradients are coordinated and can be integrated into a whole-plant conservation gradient. Leaf and root N concentrations correlated positively and were independent of phylogeny, whereas analogous leaf and root morphological traits varied independently. For abbreviations, see **Table 1**.

The results of this study provide evidence for the presence of LES and a two-dimensional RES within the community of this subtropical evergreen broad-leaved forest (**Figure 1**), which is in line with our first hypothesis. Additionally, a leaf construction trade-off axis, independent of both LES and RES, also emerged for LT-LTD (**Figure 5**).

Our leaf economics axis composed of LMA and LN was consistent with the prevailing LES found in global scale studies (Díaz et al., 2016; Wright et al., 2004) and in many regional scale studies (Xiang et al., 2013; Li et al., 2015; Hu et al., 2021; Li et al., 2022). This trait variation reflects the trade-off between resource acquisition and conservation (Reich, 2014; Díaz et al., 2016). Acquisitive species possess a high LN and low LMA, exhibit a short leaf lifespan, and demonstrate rapid growth rates. However, species with high LMA, such as the Fagaceae and Lauraceae tree species dominant in this study, exhibit slow photosynthetic efficiency and a slow growth rate. Their strategies responding to environmental stress are typically characterized by resistance and tolerance, with defense structure and extended leaf lifespan (Wright et al., 2004; Poorter et al., 2009). The variance of LMA in the present study accounted for 15% of the total global variance (**Supplementary Table S8**). Furthermore, the variance of LN in this current study was lower than that observed in any larger-scale studies (**Supplementary Table S8**), which contributed to 24% of the total global variance (**Supplementary Table S8**). This may be attributed to the effect that external environmental filtering had on species composition under similar climate and soil conditions, which resulted in narrowed variations of LMA and LN within the community.

LT and LTD are the two components of LMA, and both contribute to variation in LMA (Villar et al., 2013; Li et al., 2015). In the present study, the leaf construction axis of LT-LTD contributed more to the total variation in leaf traits than did the leaf economics axes (LMA-LN) (**Figure 2A**, **Supplementary Figure S2A**). This might be associated with the extremely significant negative correlation between LT and LTD within the community (**Supplementary Figure S7B**). Furthermore, the variances of LT and LTD in this current study was significantly higher than those observed in any larger-scale studies (**Supplementary Table S8**). Such large variations in LT and LTD within a forest community may be attributed to niche differentiation among species in response to aboveground environmental heterogeneity (especially light availability) (Shen et al., 2019; Jin et al., 2024). Plants primarily adapt to their light environment by adjusting leaf morphology and structure (Coble and Cavaleri, 2014). LT, which is determined by the number and length of palisade cell layers and is predominantly influenced by light conditions, represents a leaf’s investment in capturing light. A thicker LT is correlated with a higher photosynthetic rate per unit area of the leaf (Niinemets, 2001). Conversely, LTD is determined by cell wall thickness, cell size, and intercellular air space (Niinemets, 1999). An increase in LTD is attributed to the thickening of the cell wall and to smaller and more tightly arranged cells, which reduce the conductivity of cell CO_2_ and lower the photosynthetic potential of leaves per unit dry mass (Niinemets, 1999). LTD is also associated with leaf robustness and physical defense (Poorter et al., 2009; Méndez et al., 2019). Within a forest community, leaves in the upper part of the canopy generally exhibit thicker LT due to high light availability, which maximizes light utilization. Conversely, leaves in the lower part of the canopy display increased LTD as a result of low light availability, enabling them to acclimate to low-light conditions, to prolong leaf longevity, and to protect against insect herbivory. The study conducted by Jin et al. (2024) also showed a trade-off between LT and LTD in the *Parashorea chinensis* canopy. Such a trade-off facilitates the coexistence among species within a community as they adapt to light heterogeneity. However, this unique trade-off may not be evident on a larger scale. For instance, studies have shown that LT and LTD can vary independently at the regional scale, without exhibiting a trade-off relationship (Nadal et al., 2023). Consequently, the current study demonstrates that, while the external filtering effect of similar climatic and soil conditions results in narrowed LMA variation within a community, the heterogeneity of light availability leads to significant variation in and trade-offs between the two components of LMA (LT and LTD).

In the current study, the pPCA and PCA analyses of the five functional traits of first-order roots (**Figure 2B**; **Supplementary Figure S2B**; **Supplementary Table S3**) showed a two-dimensional RES. The first dimension, which represented the root conservation gradient, consisted of RN, RC, and RTD. Tree species with low RN and high RTD are generally considered to conserve resources more efficiently (Weemstra et al., 2016). Conversely, species with high RN and low RTD tend to prioritize resource acquisition at the expense of longevity (McCormack et al., 2012). The second dimension, which represented the collaboration gradient ranging from “do-it-yourself” (with high SRL and low RD) to “outsourcing” (with low SRL and high RD) strategies (Bergmann et al., 2020). In the present study, the arbuscular mycorrhizal (AM) species represented by Lauraceae and Magnoliaceae were generally distributed on the high RD side. In contrast to previous studies (e.g., Bergmann et al., 2020), the Fagaceae species here were mainly located at the conservative end of the conservation gradient, and shrubs were mainly distributed at the “do-it-yourself” end of the collaboration gradient (**Figure 2B**; **Supplementary Figure S2B**). The understory shrubs had lower photosynthetic rates under a lower light environment than the trees, so they could hardly afford the huge carbon cost associated with mycorrhizal symbiosis. It has been reported that mycorrhizal fungi consume up to 10% (Řezáčová et al., 2017; Řezáčová et al., 2018) and 20-50% of photosynthesis fixed C (Hobbie, 2006; Řezáčová et al., 2017; Hawkins et al., 2023) in AM and ectomycorrhizal mycorrhizae (EM) trees, respectively. Therefore, having a higher specific root length can significantly enhance soil exploration efficiency per unit carbon input and reduce reliance on mycorrhizae (Caplan et al., 2017).

Our results showed that most of the total variation (about 70%) in first-order root traits within the studied forest community could be explained by the two-dimensional RES, which was comparable to the findings of previous studies both at regional (de la Riva et al., 2021; Ding et al., 2020) and global scales (Bergmann et al., 2020; Weigelt et al., 2021; Carmona et al., 2021). This may be related to the fact that soil heterogeneity is high both at large and small scales, thus putting roots and their symbionts under similar evolutionary selection pressures at different scales. However, the relative importance of the two axes of RES in explaining total variation of root traits still differed across scales. At both the regional (de la Riva et al., 2021; Ding et al., 2020) and global scales (Bergmann et al., 2020; Weigelt et al., 2021; Carmona et al., 2021), the root collaboration gradient (SRL-RD) contributed more to total variation than did the root conservation gradient (RTD-RN), which contrasted our results within the forest community where the root conservation gradient predominated. This might be attributed to the fact that, at larger scales, the root collaboration gradient is primarily influenced by species sorting caused by environmental filtering, and can be narrowed within a forest community having similar climate and soil conditions; whereas resource heterogeneity or ecological niche differentiation may exert a greater influence on the root conservation gradient at a smaller scale within the community. Whether it was RD or SRL in the current study, the variance was much smaller compared to that of larger scales (**Supplementary Table S8**). RD and SRL accounted for 15% and 10% of the total global variance, respectively (**Supplementary Table S8**). Conversely, the variances of RTD and RN in the current study was each comparable to those of the first-order roots at the global scale (Ma et al., 2018), and accounted for 78% and 75% of the total global variance, respectively (**Supplementary Table S8**).

In our study, although the conservation gradients of roots and leaves were correlated, the CV for each root trait was higher than that for each leaf trait on the conservation gradient. This may be attributed to variation in the different spatial scales at which the driving factors influenced root and leaf traits. The driving factors influencing the leaf conservation gradient (or LES) (e.g., air temperature, precipitation, etc.) usually vary on a large scale (Poorter et al., 2009), whereas the drivers for the root conservation gradient (e.g., soil factors) are usually very heterogeneous at small scales (Defrenne et al., 2019; Weemstra et al., 2021). Hence, within a small-scale forest community, variation may be much greater in root traits than in leaf traits due to minimal variability in climatic factors and substantial variability in the soil micro-environment. This could potentially weaken the root-leaf trait correlation at small scales (e.g., within a community).

### 4.2 Covariation between analogous leaf and root traits

This study found a correlation between the axes of the leaf economics spectrum and the root conservation gradient (**Figure 3**; **Supplementary Figure S5**; **Supplementary Tables S4** and **S5**). Besides, in PCA and pPCA analysis of the six core leaf-root trait pairs, the conservation gradient axis correlated highly with LN, RN, LMA, and RTD, and orthogonally to the root collaboration gradient axis represented by SRL and RD (**Supplementary Figure S6**). These findings suggest that the conservation gradient was coordinated above and below ground and could be integrated into a whole plant conservation gradient. This finding is consistent with that of a previous global-scale study on woody plants (Weigelt et al., 2021). Thus, plant species at the “fast” end of the “whole plant conservation gradient” had both larger LN and RN, thus indicating the consistent nitrogen acquisition strategy employed by the plant’s organs. In contrast, the conservative, “slow” species are characterized by higher construction costs per unit leaf area (high LMA) and longer leaf longevity (Poorter et al., 2009), as well as by higher RTD and longer root longevity (Ryser, 1996) (**Supplementary Figure S7A**).

We found a positive correlation between LN and RN within the community of this subtropical evergreen broad-leaved forest, and it was not influenced by phylogeny (**Figure 4E**). This result is consistent with previous studies on woody plants, both at regional- and global-scales (Freschet et al., 2013; Valverde-Barrantes et al., 2015; Wang et al., 2017; Weigelt et al., 2021) and reflects the strong coordination between leaves and roots in nutrient capture and utilization, as well as in metabolism (Osunkoya et al., 2010). Plant tissue N concentration represents the metabolic activity of an organ to a certain extent (Reich et al., 2006). The high metabolic activity of roots requires high leaf photosynthesis to provide carbohydrates to roots. Conversely, the high photosynthesis of leaves requires high root metabolic activity to acquire necessary water and nutrients (Reich, 2014). This result echoes the notion that tissue nitrogen concentrations can highlight the whole plant trade-offs in intrinsic physiology and life history (Kerkhoff et al., 2006; Valverde-Barrantes et al., 2015).

In contrast to tissue nitrogen concentrations, our results showed zero or very weak correlation between analogous leaf and root morphological traits (**Figure 4**), thus supporting our second hypothesis. Previous studies have also pointed out that morphological traits exhibit decoupling patterns between aboveground and belowground organs (Fortunel et al., 2012; Yang et al., 2021), or that the correlation between analogous above and belowground morphological traits disappears after phylogenetic correction (Wang et al., 2017). Furthermore, global-scale studies have demonstrated that the correlation between analogous leaf and root traits is much weaker in morphological than in chemical traits (Weigelt et al., 2021). The decoupling between leaf and root morphological traits allows plants to independently adapt their morphology in response to diverse above- and below-ground environmental filters, thereby increasing the multitude of ecological strategies (Freschet et al., 2013; Laughlin, 2014). This may facilitate species adaptations to diverse niche dimensions, promote species coexistence and enhance ecosystem stability (Laughlin, 2014; Weigelt et al., 2021).

The decoupling between aboveground and belowground morphological traits may be attributed to multiple causes. Firstly, there are disparities between above- and below-ground organs in functional diversity, distinct selective pressures and environmental constraints (Mommer and Weemstra, 2012; Laughlin, 2014; Wang et al., 2017; Liese et al., 2017). Leaf traits are primarily driven by the main functions of light and CO_2_ capture (Poorter et al., 2009), and leaf growth and development are mainly limited by light availability and water supply. In contrast, roots are required to perform multiple functions such as water and nutrient uptake, which includes the uptake of different nutrient elements in different forms (e.g., inorganic *vs*. organic, free ion *vs*. mineral- or organic-bounded). The abiotic (e.g., soil nutrients, water, and soil structure) and biotic (soil microorganisms including mycorrhizal fungi and pathogens) stresses to which they are exposed are even more complex (Craine et al., 2005; Bardgett et al., 2014; Mccormack et al., 2015; Weemstra et al., 2016). In a forest community, leaf morphological traits may be more susceptible to microclimate conditions, such as light availability, while root morphological traits may be more sensitive to heterogeneity in soil nutrients and water (Yang et al., 2021). Therefore, different responses to the same growth environment can result in poor coordination between above- and belowground organs. Secondly, in contrast to leaves, roots can outsource the task of resource acquisition to beneficial soil microorganisms such as mycorrhizal fungi. This delegation can reduce the need to develop an acquisitive root, and the root systems of plants dependent on mycorrhizal fungi thereby undergo morphophysiological alteration (Makita et al., 2012). Thus, the coordination between aboveground and belowground morphologies may become decoupled under the influence of the root collaboration gradient. Additionally, Valverde-Barrantes et al. (2015) proposed that the decoupling between leaf-root morphological traits may be attributed to root traits having a stronger phylogenetic conservatism than leaf traits. Our results further suggested that root traits may be more phylogenetically structured than leaf traits (**Table 2**).

## 5 Conclusion

The ubiquitous LES was obvious within the community of this subtropical evergreen broad-leaved forest, but it was subordinate to an LT-LTD trade-off dimension that represents an adaptation of leaf construction to light heterogeneity. Similar to large-scale studies, the two-dimensional RES, meaning the root conservation and root collaboration gradients, dominated the total variation of root traits within this forest community, thereby implying that roots face similar selection pressures and adopt similar evolutionary adaptation strategies across different spatial scales. The LES correlated with the root conservation gradient, and both could be integrated into a whole plant conservation gradient independent of the root collaboration gradient and the leaf LT-LTD trade-off dimension. Thus, a single axis of the plant economics spectrum fails to fully capture the variation in below- and aboveground plant traits (**Figure 5**). Leaves and first-order roots integrated their analogous morphological and chemical traits differently. Leaf and root N concentrations were highly correlated and were not affected by phylogeny. The analogous pairs of aboveground and belowground morphological traits were decoupled. Such trait multidimensionality and complex ecological linkages between above- and below-ground traits in plants highlights their ability to exhibit diverse whole-plant adaptation strategies in which they modify the acquisition of above- and below-ground resources and enhance their capacity for environmental adaptation. These findings provide within-community evidence for a multidimensional view of plant functional traits, and offer important insights into ecological trade-offs, species coexistence and community assembly in forest ecosystems.

## Data Availability

The datasets presented in this study can be found in online repositories. The names of the repository/repositories and accession number(s) can be found in the article/**Supplementary Material**.
