# Supplementary material for: Does a whole plant conservation gradient exist within a subtropical broadleaved evergreen forest?
Source: Front Plant Sci. 2024 Dec 11;15:1481323. doi: 10.3389/fpls.2024.1481323 (PMC11668598; doi:10.3389/fpls.2024.1481323)
Supplement: Supplementary file 1 [file DataSheet1.docx]

Supplementary Material

# Supplementary Tables and Figures

## Supplementary Tables

**Table S1** Tree species sampled in the studied subtropical broadleaved evergreen forest.

| No. | Species | Family | Genus | Growth form |
| --- | --- | --- | --- | --- |
| 1 | *Alniphyllum fortunei* | Styracaceae | *Alniphyllum* | Tree |
| 2 | *Styrax suberifolius* | Styracaceae | *Styrax* | Tree |
| 3 | *Maesa japonica* | Primulaceae | *Maesa* | Shrub |
| 4 | *Gmelina chinensis* | Lamiaceae | *Gmelina* | Tree |
| 5 | *Vernicia fordii* | Euphorbiaceae | *Vernicia* | Tree |
| 6 | *Ilex chinensis* | Aquifoliaceae | *Ilex* | Shrub |
| 7 | *Ilex rotunda* | Aquifoliaceae | *Ilex* | Shrub |
| 8 | *Ilex triflora* | Aquifoliaceae | *Ilex* | Tree |
| 9 | *Ilex tutcheri* | Aquifoliaceae | *Ilex* | Tree |
| 10 | *Elaeocarpus chinensis* | Elaeocarpaceae | *Elaeocarpus* | Tree |
| 11 | *Elaeocarpus decipiens* | Elaeocarpaceae | *Elaeocarpus* | Tree |
| 12 | *Elaeocarpus japonicus* | Elaeocarpaceae | *Elaeocarpus* | Tree |
| 13 | *Sloanea sinensis* | Elaeocarpaceae | *Sloanea* | Tree |
| 14 | *Daphniphyllum oldhamii* | Daphniphyllaceae | *Daphniphyllum* | Tree |
| 15 | *Distyliopsis dunnii* | Hamamelidaceae | *Distyliopsis* | Shrub |
| 16 | *Loropetalum chinense* | Hamamelidaceae | *Loropetalum* | Shrub |
| 17 | *Castanopsis carlesii* | Fagaceae | *Castanopsis* | Tree |
| 18 | *Castanopsis faberi* | Fagaceae | *Diospyros* | Tree |
| 19 | *Castanopsis jucunda* | Fagaceae | *Castanopsis* | Tree |
| 20 | *Castanopsis lamontii* | Fagaceae | *Castanopsis* | Tree |
| 21 | *Castanopsis sclerophylla* | Fagaceae | *Castanopsis* | Tree |
| 22 | *Cyclobalanopsis glauca* | Fagaceae | *Cyclobalanopsis* | Tree |
| 23 | *Lithocarpus polystachyus* | Fagaceae | *Lithocarpus* | Tree |
| 24 | *Michelia fujianensis* | Magnoliaceae | *Michelia* | Tree |
| 25 | *Michelia odora* | Magnoliaceae | *Michelia* | Tree |
| 26 | *Michelia skinneriana* | Magnoliaceae | *Michelia* | Tree |
| 27 | *Rhus chinensis* | Anacardiaceae | *Rhus* | Shrub |
| 28 | *Aidia cochinchinensis* | Rubiaceae | *Diplospora* | Tree |
| 29 | *Diplospora dubia* | Rubiaceae | *Randia* | Shrub |
| 30 | *Photinia bodinieri* | Rosaceae | *Photinia* | Tree |
| 31 | *Photinia prunifolia* | Rosaceae | *Photinia* | Tree |
| 32 | *Ficus erecta* | Moraceae | *Ficus* | Shrub |
| 33 | *Schima superba* | Theaceae | *Schima* | Tree |
| 34 | *Symplocos fukienensis* | Symplocaceae | *Symplocos* | Tree |
| 35 | *Symplocos glauca* | Symplocaceae | *Symplocos* | Tree |
| 36 | *Symplocos lancifolia* | Symplocaceae | *Symplocos* | Tree |
| 37 | *Symplocos oblanceolata* | Symplocaceae | *Symplocos* | Tree |
| 38 | *Alangium chinense* | Cornaceae | *Alangium* | Tree |
| 39 | *Diospyros kaki* var. *silvestris* | Ebenaceae | *Diospyros* | Tree |
| 40 | *Diospyros oleifera* | Ebenaceae | *Diospyros* | Tree |
| 41 | *Itea omeiensis* | Iteaceae | *Itea* | Shrub |
| 42 | *Ziziphus jujuba* var. *spinosa* | Rhamnaceae | *Ziziphus* | Shrub |
| 43 | *Dendropanax dentiger* | Araliaceae | *Dendropanax* | Shrub |
| 44 | *Heteropanax chinensis* | Araliaceae | *Heteropanax* | Tree |
| 45 | *Adinandra milletii* | Pentaphylacaceae | *Adinandra* | Shrub |
| 46 | *Eurya loquaiana* | Pentaphylacaceae | *Eurya* | Shrub |
| 47 | *Altingia gracilipes* | Altingiaceae | *Altingia* | Tree |
| 48 | *Liquidambar formosana* | Altingiaceae | *Liquidambar* | Tree |
| 49 | *Antidesma bunius* | Phyllanthaceae | *Antidesma* | Shrub |
| 50 | *Phyllanthus glaucus* | Phyllanthaceae | *Phyllanthus* | Tree |
| 51 | *Cinnamomum austrosinense* | Lauraceae | *Cinnamomum* | Tree |
| 52 | *Cinnamomum camphora* | Lauraceae | *Cinnamomum* | Tree |
| 53 | *Cinnamomum japonicum* | Lauraceae | *Cinnamomum* | Tree |
| 54 | *Cinnamomum micranthum* | Lauraceae | *Cinnamomum* | Tree |
| 55 | *Lindera communis* | Lauraceae | *Lindera* | Tree |
| 56 | *Litsea subcoriacea* | Lauraceae | *Litsea* | Tree |
| 57 | *Machilus chrysotricha* | Lauraceae | *Machilus* | Tree |
| 58 | *Machilus pauhoi* | Lauraceae | *Machilus* | Tree |
| 59 | *Neolitsea aurata* | Lauraceae | *Neolitsea* | Tree |
| 60 | *Phoebe bournei* | Lauraceae | *Phoebe* | Tree |

**Table S2** Results of principal component analysis (PCA) and phylogenetic principal component analysis (pPCA) of leaf traits in 60 tree species in the subtropical evergreen broad-leaved forest. All trait data was log_10_­transformed prior to analysis. Trait abbreviations: LT, leaf thickness; LMA, specific leaf mass; LTD, leaf tissue density; LC, leaf carbon concentration; LN, leaf nitrogen concentration. RD, root diameter; SRL, specific root length; RTD, root tissue density; RC, root carbon concentration; RN, root nitrogen concentration.

| PCA | | | | | | pPCA | | | | |  |
| --- | --- | --- | --- | --- | --- | --- | --- | --- | --- | --- | --- |
| Variable | PC1 | PC2 | PC3 | PC4 | PC5 | pPC1 | pPC2 | pPC3 | pPC4 | pPC5 | |
| Eigenvalue | 2.014 | 1.513 | 0.89 | 0.553 | 0.031 | 2.038 | 1.527 | 0.872 | 0.541 | 0.023 | |
| Variation  explained (%) | 40.27 | 30.26 | 17.79 | 11.06 | 0.61 | 40.75 | 30.53 | 17.44 | 10.83 | 0.45 | |
| LT | **-0.749** | **0.631** | -0.136 | 0.099 | 0.113 | **-0.826** | -0.505 | 0.214 | 0.087 | 0.099 | |
| LMA | **0.562** | **0.603** | -0.248 | 0.507 | -0.046 | 0.500 | **-0.656** | 0.277 | 0.492 | -0.037 | |
| LTD | **0.962** | -0.242 | 0.027 | 0.024 | 0.124 | **0.977** | 0.150 | -0.106 | 0.036 | 0.106 | |
| LC | 0.148 | 0.489 | **0.859** | -0.005 | -0.005 | -0.075 | **-0.544** | **-0.834** | 0.050 | -0.003 | |
| LN | -0.435 | **-0.674** | 0.266 | 0.535 | 0.017 | -0.383 | **0.724** | -0.205 | 0.536 | 0.011 | |

**Table S3** Results of principal component analysis (PCA) and phylogenetic principal component analysis (pPCA) of first-order root traits in 60 tree species in the subtropical evergreen broad-leaved forest. All trait data was log_10_­transformed prior to analysis. Trait abbreviations: RD, root diameter; SRL, specific root length; RTD, root tissue density; RC, root carbon concentration; RN, root nitrogen concentration.

| PCA | | | | | | pPCA | | | | |  |
| --- | --- | --- | --- | --- | --- | --- | --- | --- | --- | --- | --- |
| Variable | PC1 | PC2 | PC3 | PC4 | PC5 | pPC1 | pPC2 | pPC3 | pPC4 | pPC5 | |
| Eigenvalue | 1.915 | 1.585 | 0.859 | 0.597 | 0.044 | 1.943 | 1.470 | 0.916 | 0.621 | 0.050 | |
| Variation  Explained (%) | 38.30 | 31.71 | 17.18 | 11.93 | 0.88 | 38.85 | 29.41 | 18.32 | 12.42 | 1.00 | |
| RD | 0.412 | **0.845** | 0.213 | -0.239 | 0.117 | 0.324 | **0.888** | -0.259 | 0.155 | -0.122 | |
| SRL | 0.495 | **-0.803** | 0.257 | 0.17 | 0.122 | **0.587** | **-0.745** | -0.248 | -0.149 | -0.131 | |
| RTD | **-0.877** | -0.026 | -0.463 | 0.03 | 0.124 | **-0.862** | -0.086 | 0.480 | 0.017 | -0.134 | |
| RC | **-0.596** | 0.318 | **0.562** | 0.478 | 0.004 | **-0.643** | 0.159 | **-0.508** | **-0.550** | -0.002 | |
| RN | **0.614** | 0.352 | -0.466 | **0.53** | 0.003 | **0.580** | 0.307 | **0.546** | **-0.521** | -0.001 | |

**Table S4** Results of principal component analysis of 10 functional traits in 60 tree species in the subtropical evergreen broad-leaved forest. All trait data was log10­transformed prior to analysis. Trait abbreviations are given in Table S2 and Table S3.

|  | PC1 | PC2 | PC3 | PC4 | PC5 | PC6 | PC7 | PC8 | PC9 | PC10 |
| --- | --- | --- | --- | --- | --- | --- | --- | --- | --- | --- |
| Eigenvalue | 2.589 | 2.064 | 1.704 | 1.096 | 0.884 | 0.653 | 0.546 | 0.397 | 0.043 | 0.023 |
| Variation explained (%) | 25.89 | 20.64 | 17.04 | 10.96 | 8.84 | 6.53 | 5.46 | 3.97 | 0.43 | 0.23 |
| LT | 0.324 | **-0.87** | 0.087 | -0.133 | 0.236 | -0.196 | 0.084 | -0.016 | 0.034 | 0.097 |
| LMA | **0.554** | 0.41 | 0.105 | -0.403 | 0.287 | -0.313 | 0.365 | 0.191 | -0.014 | -0.038 |
| LTD | 0.017 | **0.972** | -0.05 | -0.116 | -0.051 | 0.136 | -0.022 | 0.081 | 0.032 | 0.102 |
| LC | 0.396 | 0.112 | 0.296 | **0.517** | **0.602** | 0.132 | -0.251 | 0.184 | -0.002 | -0.007 |
| LN | **-0.675** | -0.222 | 0.31 | 0.304 | -0.231 | 0.013 | 0.265 | 0.428 | -0.007 | 0.014 |
| RD | -0.379 | 0.006 | **0.782** | -0.416 | 0.004 | -0.042 | -0.238 | 0.024 | 0.113 | -0.025 |
| SRL | -0.424 | -0.108 | **-0.795** | -0.078 | 0.316 | 0.188 | 0.114 | 0.092 | 0.117 | -0.022 |
| RTD | **0.752** | 0.100 | 0.031 | 0.488 | -0.361 | -0.154 | 0.12 | -0.052 | 0.12 | -0.014 |
| RC | **0.571** | -0.231 | 0.312 | -0.209 | -0.073 | **0.648** | 0.233 | -0.014 | -0.001 | -0.008 |
| RN | **-0.592** | 0.239 | 0.399 | 0.287 | 0.305 | -0.002 | 0.365 | -0.354 | 0.01 | 0.007 |

**Table S5** Results of phylogenetic principal component analysis of 10 functional traits in 60 tree species in subtropical evergreen broad-leaved forest. All trait data was log10­transformed prior to analysis. Trait abbreviations are given in Table S2 and Table S3.

|  | PC1 | PC2 | PC3 | PC4 | PC5 | PC6 | PC7 | PC8 | PC9 | PC10 |
| --- | --- | --- | --- | --- | --- | --- | --- | --- | --- | --- |
| Eigenvalue | 0.401 | **-0.841** | 0.059 | 0.145 | -0.193 | 0.239 | 0.055 | -0.027 | -0.027 | 0.096 |
| Variation explained (%) | **0.497** | 0.452 | -0.079 | 0.416 | -0.362 | 0.306 | 0.296 | 0.237 | 0.010 | -0.035 |
| LT | 0.401 | **-0.841** | 0.059 | 0.145 | -0.193 | 0.239 | 0.055 | -0.027 | -0.027 | 0.096 |
| LMA | **0.497** | 0.452 | -0.079 | 0.416 | -0.362 | 0.306 | 0.296 | 0.237 | 0.010 | -0.035 |
| LTD | -0.103 | **0.961** | -0.054 | 0.085 | 0.009 | -0.194 | 0.005 | 0.086 | -0.020 | 0.100 |
| LC | 0.479 | 0.013 | -0.112 | **-0.537** | **-0.587** | -0.212 | -0.264 | 0.100 | 0.003 | -0.006 |
| LN | **-0.648** | -0.333 | -0.290 | -0.317 | 0.129 | 0.060 | 0.110 | **0.502** | 0.011 | 0.012 |
| RD | -0.380 | -0.151 | **-0.757** | 0.425 | -0.145 | -0.106 | -0.170 | -0.036 | -0.126 | -0.016 |
| SRL | -0.425 | -0.081 | **0.845** | 0.048 | -0.187 | -0.151 | 0.103 | 0.101 | -0.132 | -0.015 |
| RTD | **0.735** | 0.228 | -0.145 | -0.451 | 0.334 | 0.223 | 0.053 | 0.007 | -0.139 | -0.008 |
| RC | **0.609** | -0.330 | -0.177 | 0.088 | 0.119 | **-0.554** | 0.398 | 0.037 | 0.004 | -0.005 |
| RN | **-0.618** | 0.113 | -0.262 | -0.413 | -0.275 | 0.094 | 0.442 | -0.293 | -0.008 | 0.007 |

**Table S6** Akaike’s information criterion (AIC) values for trait relationships between leaves and absorptive roots with different phylogenetic correlation structures and generalized least squares (GLS). Models with the lowest AIC values were selected as the best phylogenetic correlation structure for phylogenetic generalized least squares (PGLS) analyses; the best models are bolded. Brownian, Brownian correlation structure; Martin’s, Martin’s correlation structure; Pagel’s, Pagel’s correlation structure. Trait abbreviations are given in Table S2 and Table S3.

|  | Brownian | Martin’s | Pagel’s | GLS |
| --- | --- | --- | --- | --- |
| RD-LT | 93.65 | -87.00 | **-90.65** | -88.27 |
| SRL-LMA | 77.39 | 6.34 | **0.74** | 4.34 |
| RTD-LTD | 96.42 | 1.93 | **-1.33** | 0.78 |
| RC-LC | -213.13 | -279.26 | **-279.39** | -280.67 |
| RN-LN | 105.31 | -47.84 | **-60.32** | -49.84 |

**Table S7** Model outputs for generalized least squares (GLS) and phylogenetic generalized least squares (PGLS) regressions of the leaf-root trait relationships in 60 tree species in subtropical evergreen broad-leaved forest. Abbreviations: λ, Pagel’s lambda; Log-lik, log likelihood. Statistically significant relationships are in bold (*p* < 0.05). All trait data was log_10_-transformed prior to analysis. Trait abbreviations are given in Table S2 and Table S3.

|  | | Slope | Intercept | *R^2^* | *p* | λ | Log-Lik |
| --- | --- | --- | --- | --- | --- | --- | --- |
| RD-LT | GLS | -0.007 | -0.45 | <0.001 | 0.913 |  |  |
|  | PGLS | -0.002 | -0.45 | <0.001 | 0.971 | 0.24 | 49.33 |
| SRL-LMA | GLS | -0.49 | 2.56 | 0.06 | 0.064 |  |  |
|  | PGLS | -0.46 | 2.52 | 0.02 | 0.064 | 0.70 | 3.63 |
| RTD-LTD | GLS | 0.04 | -0.53 | 0.002 | 0.735 |  |  |
|  | PGLS | 0.06 | -0.54 | 0.01 | 0.562 | 0.76 | 4.67 |
| RC-LC | GLS | 0.11 | 2.35 | 0.03 | 0.208 |  |  |
|  | PGLS | 0.12 | 2.30 | 0.01 | 0.176 | 0.18 | 143.69 |
| RN-LN | GLS | 0.73 | 0.20 | 0.19 | **<0.001** |  |  |
|  | PGLS | 0.68 | 0.27 | 0.22 | **<0.001** | 0.41 | 34.16 |

**Table S8** Comparisons of plant traits among different studies. Trait abbreviations are given in Table S2 and Table S3.

| Trait | Mean | Range | Variance | Species (n) | Study region | Root group | Reference |
| --- | --- | --- | --- | --- | --- | --- | --- |
| LT (mm) | 0.4 | 0.12~1.16 | 0.06 | Woody (60) | Subtropical forest (within community) |  | This study |
|  | 0.24 | 0.13~0.43 | 0.003 | Woody (84) | Tropical cloud forest (within community) |  | (Yang et al., 2021) |
|  | 0.17 | 0.07~0.35 | 0.003 | Seedling (57) | Subtropical forest (different altitudes) |  | (Shen et al., 2019) |
|  | 0.24 | 0.09~0.56 | 0.007 | Woody (85) | Tropical-subtropical forests |  | (Li et al., 2015) |
|  | 0.18 |  | 0.012 | Woody (112) | Subtropical and temperate forests |  | (Wang et al., 2017) |
|  | 0.24 |  |  | (1590) | Global |  | (Weigelt et al., 2021) |
|  | 0.28 | 0.007~3.74 | 0.05 | (2600) | Global |  | TRY (Kattge et al., 2020) |
| LMA (g·m^-2^) | 116.22 | 52~217 | 1085.26 | Woody (60) | Subtropical forest (within community) |  | This study |
|  | 100 | 38~173 | 729 | Woody (84) | Tropical cloud forest (within community) |  | (Yang et al., 2021) |
|  | 49.3 | 23.4~128 | 350.96 | Seedling (57) | Subtropical forest (different altitudes) |  | (Shen et al., 2019) |
|  | 80 | 33~159 | 739.84 | Woody (85) | Tropical-subtropical forests |  | (Li et al., 2015) |
|  | 64.02 |  | 1313 | Woody (112) | Subtropical and temperate forests |  | (Wang et al., 2017) |
|  | 60 |  |  | (3989) | Global |  | (Weigelt et al., 2021) |
|  | 70.29 | 6.8~3333 | 7163.5 | (3761) | Global |  | TRY (Kattge et al., 2020) |
| LTD (g cm^-3^) | 0.38 | 0.09~0.98 | 0.05 | Woody (60) | Subtropical forest (within community) |  | This study |
|  | 0.34 | 0.14~0.86 | 0.013 | Woody (85) | Tropical-subtropical forests |  | (Li et al., 2015) |
|  | 0.52 |  | 0.036 | Woody (112) | Subtropical and temperate forests |  | (Wang et al., 2017) |
|  | 0.33 |  |  | (1652) | Global |  | (Weigelt et al., 2021) |
|  | 0.33 | 0.04~1.31 | 0.002 | (1764) | Global |  | TRY (Kattge et al., 2020) |
| LC (mg g^-1^) | 451.67 | 356.85~509.5 | 1239.48 | Woody (60) | Subtropical forest (within community) |  | This study |
|  | 406.4 | 334.2~470.5 | 809.29 | Seedling (57) | Subtropical forest (different altitudes) |  | (Shen et al., 2019) |
|  | 473.56 |  | 807.33 | Woody (112) | Subtropical and temperate forests |  | (Wang et al., 2017) |
|  | 470.63 | 330~623.8 | 1462.19 |  | Global |  | TRY (Kattge et al., 2020) |
| LN (mg·g^-1^) | 17.44 | 10.76~33.49 | 18.26 | Woody (60) | Subtropical forest (within community) |  | This study |
|  | 11.83 | 7.48~30.93 | 11.37 | Woody (84) | Tropical cloud forest (within community) |  | (Yang et al., 2021) |
|  | 18.05 | 10.72~40.14 | 27.39 | Seedling (57) | Subtropical forest (different altitudes) |  | (Shen et al., 2019) |
|  | 18.05 | 5.40~40.69 | 49.55 | Woody (85) | Tropical-subtropical forests |  | (Li et al., 2015) |
|  | 22.62 |  | 55.72 | Woody (112) | Subtropical and temperate forests |  | (Wang et al., 2017) |
|  | 19.24 |  |  | (3259) | Global |  | (Weigelt et al., 2021) |
|  | 20.42 | 2.37~68.98 | 75.22 | 6405 | Global |  | TRY (Kattge et al., 2020) |
| RD (mm) | 0.36 | 0.19~0.63 | 0.009 | Woody (60) | Subtropical forest (within community) | First-order root | This study |
|  | 0.34 | 0.23~0.59 | 0.012 | Dominant  woody (20) | Subtropical forest (within community) | Intact root branch | (Han et al., 2022) |
|  | 0.54 | 0.33~0.99 | 0.018 | Seedling (57) | Subtropical forest (different altitudes) | Fine root | (Shen et al., 2019) |
|  | 0.36 | 0.23~0.67 | 0.009 | Woody (69) | Temperate forest (different altitudes) | Absorptive root | (Wang et al., 2024) |
|  | 0.34 | 0.07~1.01 | 0.039 | Woody (96) | Tropical-subtropical forests | First-order root | (Kong et al., 2014) |
|  | 0.28 | 0.12~0.68 | 0.014 | Woody (135) | Subtropical and boreal forests | First-order root | (Wang et al., 2018) |
|  | 0.29 | 0.08~1.01 | 0.027 | (369) | Global | First-order root | (Ma et al., 2018) |
|  | 0.38 |  |  | (1773) | Global |  | (Weigelt et al., 2021) |
|  | 0.40 | 0.03~1.92 | 0.059 | (1143) | Global | Fine root | TRY (Kattge et al., 2020) |
| SRL (m·g^-1^) | 43.69 | 13.12~190.13 | 1075.53 | Woody (60) | Subtropical forest (within community) | First-order root | This study |
|  | 56.76 | 15.96~109.39 | 734.95 | Dominant  woody (20) | Subtropical forest (within community) | Intact root branch | (Han et al., 2022) |
|  | 5.59 | 1.90~15.24 | 687.55 | Woody (84) | Tropical cloud forest (within community) | Fine root | (Yang et al., 2021) |
|  | 15.69 | 5.41~38.36 | 49.85 | Seedling (57) | Subtropical forest (different altitudes | Fine root | (Shen et al., 2019) |
|  | 32.42 | 10.80~60.99 | 159.87 | Woody (69) | Temperate forest (different altitudes) | Absorptive root | (Wang et al., 2024) |
|  | 56.19 | 4.42~169.91 | 1481.49 | Woody (96) | Tropical-subtropical forests | First-order root | (Kong et al., 2014) |
|  | 109.37 | 10.73~580.27 | 6012.97 | Woody (135) | Subtropical and boreal forests | First-order root | (Wang et al., 2018) |
|  | 110.9 | 4.4~657.1 | 14080.91 | (323) | Global | First-order root | (Ma et al., 2018) |
|  | 38.35 |  |  | (2242) | Global |  | (Weigelt et al., 2021) |
|  | 69.67 | 0.07~914 | 8981.13 | (1304) | Global | Fine root | TRY (Kattge et al., 2020) |
| RTD (g·cm^-3^) | 0.29 | 0.07~0.83 | 0.029 | Woody (60) | Subtropical forest (within community) | First-order root | This study |
|  | 0.27 | 0.16~0.34 | 0.002 | Dominant  woody (20) | Subtropical forest (within community) | Intact root branch | (Han et al., 2022) |
|  | 0.49 | 0.07~1.14 | 0.036 | Woody (84) | Tropical cloud forest (within community) | Fine root | (Yang et al., 2021) |
|  | 0.36 | 0.21~0.52 | 0.003 | Woody (69) | Temperate forest (different altitudes) | Absorptive root | (Wang et al., 2024) |
|  | 0.40 | 0.16~0.67 | 0.016 | Seedling (57) | Subtropical forest (different altitudes) | Fine root | (Shen et al., 2019) |
|  | 0.35 | 0.13~0.72 | 0.018 | Woody (96) | Tropical-subtropical forests | First-order root | (Kong et al., 2014) |
|  | 0.23 |  | 0.005 | Woody (112) | Subtropical and temperate forests | First-order root | (Wang et al., 2017) |
|  | 0.32 | 0.06~0.95 | 0.028 | (315) | Global | First-order root | (Ma et al., 2018) |
|  | 0.18 |  |  | (1633) | Global |  | (Weigelt et al., 2021) |
|  | 0.30 | 0.03~1.71 | 0.038 | (878) | Global | Fine root | TRY (Kattge et al., 2020) |
| RC (mg·g^-1^) | 433.76 | 346.3~486.1 | 488.71 | Woody (60) | Subtropical forest (within community) | First-order root | This study |
|  | 502.88 | 470.16~545.97 | 337.09 | Dominant  woody (20) | Subtropical forest (within community) | Intact root branch | (Han et al., 2022) |
|  | 466.8 | 323.4~590.1 | 2092.73 | Woody (96) | Tropical-subtropical forests | First-order root | (Kong et al., 2014) |
|  | 518.89 | 359.86~751.16 | 4907.02 | Woody (135) | Subtropical and boreal forests | First-order root | (Wang et al., 2018) |
|  | 473.0 | 307.7~684.4 | 3221.70 | (252) | Global | First-order root | (Ma et al., 2018) |
|  | 448.96 | 222.66~590.06 | 1964.69 | 436 | Global | Fine root | TRY (Kattge et al., 2020) |
| RN (mg·g^-1^) | 13.78 | 5.54~30.19 | 30.83 | Woody (60) | Subtropical forest (within community) | First-order root | This study |
|  | 17.77 | 8.96~30.39 | 29.16 | Dominant  woody (20) | Subtropical forest (within community) | Intact root branch | (Han et al., 2022) |
|  | 28.9 | 14.7~56.5 | 102.31 | Woody (84) | Tropical cloud forest (within community) | Fine root | (Yang et al., 2021) |
|  | 12.95 | 2.27~30.24 | 29.58 | Seedling (57) | Subtropical forest (different altitudes | Fine root | (Shen et al., 2019) |
|  | 14.24 | 5.89~34.05 | 23.44 | Woody (69) | Temperate forest (different altitudes) | Absorptive root | (Wang et al., 2024) |
|  | 19.38 | 5.55~44.62 | 38.22 | Woody (96) | Tropical-subtropical forests | First-order root | (Kong et al., 2014) |
|  | 19.365 | 10.03~39.93 | 30.89 | Woody (135) | Subtropical and boreal forests | First-order root | (Wang et al., 2018) |
|  | 19.3 | 5.6~50.3 | 33.52 | (284) | Global | First-order root | (Ma et al., 2018) |
|  | 10.83 |  |  | (2004) | Global |  | (Weigelt et al., 2021) |
|  | 13.62 | 1.92~44.62 | 41.29 | (780) | Global | Fine root | TRY (Kattge et al., 2020) |

## Supplementary Figures

**Figure S1** Phylogenetic tree of 60 tree species in a subtropical evergreen broad-leaved forest. Trait values are standardized. Symbol size indicates trait value after standardization for each species. Smaller symbols signify a value closer to the mean, black symbols represent values above the mean and white symbols represent values below the mean.

**Figure S2** Principal component analysis (PCA) (leaves (A) and first-order roots (B)) of functional traits in 60 woody species in a subtropical evergreen broad-leaved forest.

**Figure S3** Differences in the tree and shrub scores along the leaf and first-order root pPCA axes. ^*^, *p* < 0.05; ^**^, *p* < 0.01; ^***^, *p* < 0.001; ns, not significant.

**Figure S4** Differences in the tree and shrub leaf and first-order root traits. ^*^, *p* < 0.05; ^**^, *p* < 0.01; ^***^, *p* < 0.001; ns, not significant.

**Figure S5** Principal component analysis (PCA) for 10 leaf and first-order root traits of 60 tree species in the subtropical evergreen broad-leaved forest.

**Figure S6** Principal component analysis (PCA) (A) and phylogenetic principal component analysis (pPCA) (B) for 6 core leaf and first-order root traits of 60 tree species in the subtropical evergreen broad-leaved forest.

**Figure S7** Relationships between leaf and first order root traits of 60 tree species in the subtropical evergreen broad-leaved forest, calculated using generalized least squares (GLS) and phylogenetic generalized least squares (PGLS) methods.

**References**

Han, M., Chen, Y., Li, R., Yu, M., Fu, L., Li, S., et al. (2022). Root phosphatase activity aligns with the collaboration gradient of the root economics space. *New Phytol.* 234, 837–849. doi: 10.1111/nph.17906

Kattge, J., Bönisch, G., Díaz, S., Lavorel, S., Prentice, I. C., Leadley, P., et al. (2020). TRY plant trait database – enhanced coverage and open access. *Glob. Chang. Biol.* 26, 119–188. doi: 10.1111/gcb.14904

Kong, D., Ma, C., Zhang, Q., Li, L., Chen, X., Zeng, H., et al. (2014). Leading dimensions in absorptive root trait variation across 96 subtropical forest species. *New Phytol.* 203, 863–872. [doi: 10.1111/nph.12842](https://doi.org/10.1111/nph.12842)

Li, L., McCormack, M. L., Ma, C., Kong, D., Zhang, Q., Chen, X., et al. (2015). Leaf economics and hydraulic traits are decoupled in five species-rich tropical-subtropical forests. *Ecol. Lett.* 18, 899–906. doi: 10.1111/ele.12466

Ma, Z., Guo, D., Xu, X., Lu, M., Bardgett, R. D., Eissenstat, D. M., et al. (2018). Evolutionary history resolves global organization of root functional traits. *Nature* 555, 94–97. doi: 10.1038/nature25783

Shen, Y., Gilbert, G. S., Li, W., Fang, M., Lu, H., and Yu, S. (2019). Linking Aboveground Traits to Root Traits and Local Environment: Implications of the Plant Economics Spectrum. *Front. Plant Sci.* 10:1412. doi: 10.3389/fpls.2019.01412

Wang, R., Wang, Q., Zhao, N., Xu, Z., Zhu, X., Jiao, C., et al. (2018b). Different phylogenetic and environmental controls of first-order root morphological and nutrient traits: Evidence of multidimensional root traits. *Funct. Ecol.* 32, 29–39. doi: 10.1111/1365-2435.12983

Wang, R., Wang, Q., Zhao, N., Yu, G., and He, N. (2017). Complex trait relationships between leaves and absorptive roots: Coordination in tissue N concentration but divergence in morphology. *Ecol. Evol.* 7, 2697–2705. doi: 10.1002/ece3.2895

Wang, X., Liu, X., Mo, W., Chen, K., Chen, H., Gao, H., et al. (2024). Do phylogenetic and environmental factors drive the altitudinal variation in absorptive root traits at the species and community levels? *Plant Soil* 494, 203–215. doi: 10.1007/s11104-023-06267-1

Weigelt, A., Mommer, L., Andraczek, K., Iversen, C. M., Bergmann, J., Bruelheide, H., et al. (2021). An integrated framework of plant form and function: the belowground perspective. *New Phytol.* 232, 42–59. doi: 10.1111/nph.17590

Yang, Y., Xiao, C., Wu, X., Long, W., Feng, G., and Liu, G. (2021). Differing Trade-Off Patterns of Tree Vegetative Organs in a Tropical Cloud Forest. *Front. Plant Sci.* 12: 680379. doi: 10.3389/fpls.2021.68
